# Factors influencing time spent in hospital for unscheduled readmissions after stroke discharge

**DOI:** 10.1177/17474930251355864

**Published:** 2025-06-25

**Authors:** Melanie Turner, David J McLernon, Peter Murchie, Peter Langhorne, Mary-Joan Macleod

**Affiliations:** 1 Institute of Applied Health Sciences, School of Medicine, Medical Sciences and Nutrition, University of Aberdeen, Aberdeen, UK; 2 School of Cardiovascular and Metabolic Health, University of Glasgow, Glasgow, UK; 3 Institute of Medical Sciences, School of Medicine, Medical Sciences and Nutrition, University of Aberdeen, Aberdeen, UK

**Keywords:** Stroke, unscheduled admission, readmission, length of hospital stay

## Abstract

**Background and Aims:**

Unscheduled hospital readmission following stroke is common and has a significant effect on quality of life for patients and their families. However, there is limited evidence on the factors associated with unscheduled hospital readmission time in the first-year post-stroke discharge. This study aims to investigate patient and healthcare system factors associated with unscheduled hospital readmission time in a cohort of first-ever stroke patients in Scotland, UK.

**Methods:**

This is a population-level data-linkage study using data on adult stroke patients admitted to hospital between 2010 and 2018, with follow-up to end of 2019. The association between unscheduled hospital readmission time and patient and healthcare system factors was assessed using multivariable zero-inflated negative binomial estimations.

**Results:**

Among the 48,040 stroke patients (median age 73 years [interquartile range (IQR) 63–82]; 48.7% female) included in the study, 14,794 (30.8%) had at least one unscheduled readmission in the 1-year post-stroke discharge follow-up (median age 76 years [IQR 66–83]; 51.5% female). Median time spent in hospital as an unscheduled readmission in the 1-year follow-up was 9 days [IQR 3–25]. After adjustment, an increased risk of total unscheduled readmission time was associated with increasing age (≥ 80 years versus < 50 years Incidence Rate Ratio (IRR) 2.23 (95% CI 1.96–2.53)); living alone before stroke (IRR 1.17 (95% CI 1.11–1.24)); stroke severity (most versus least severe IRR 1.14 (95% CI 1.04–1.26)); intracerebral hemorrhage (IRR 1.29 (95% CI 1.18–1.42)); higher Charlson Comorbidity Index (CCI) (⩾3 versus 0 IRR 1.17 (95% CI 1.08–1.26)); higher frailty index (severe versus none IRR 1.16 (1.01–1.35); and longer length of stay for initial stroke admission (>10 days IRR 1.28 (95% CI 1.21–1.36)). Reduced risk of unscheduled readmission time was associated with lower socio-economic deprivation (least versus most deprived IRR 0.91 (95% CI 0.83–0.99)); prior transient ischaemic attack (TIA) (IRR 0.85 (95% CI 0.75–0.96)); and receipt of complete stroke care bundle (IRR 0.94 (95% CI 0.88–0.99)).

**Conclusion:**

Increased unscheduled hospital readmission time was associated with several factors including living alone, a higher comorbidity burden, stroke severity, and stroke type. Greater community support for these at-risk patients in terms of living arrangements and more pro-active outpatient management of comorbidities may be needed to reduce unscheduled readmission time following stroke discharge.

## Graphical abstract

**Graphical abstract:**
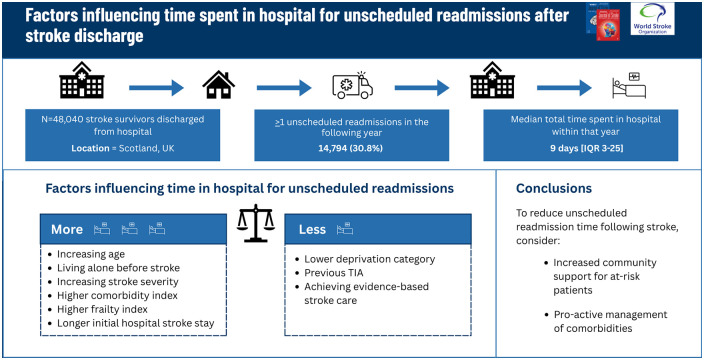

## Introduction

Despite global population growth and higher life expectancies resulting in over 12.2 million new strokes worldwide each year,^1^ major improvements in acute stroke treatment and secondary prevention mean mortality from stroke has declined substantially.^2,3^ However, these decreased mortality rates mean that many stroke patients require hospital readmission and substantial further care.^4^ Hospital readmission has been found to be particularly prevalent in the first year following a stroke, with 30-day average estimates of 17.4% (95% confidence interval [CI] 12.7–23.5%), and 1-year average estimates of 42.5% (95% CI, 34.1–51.3%), with recurrent stroke, vascular events, infections, and fractures being the most frequent reasons for readmission.^5^ Stroke therefore represents a major health burden on individuals and health and social care systems beyond the initial stroke admission.

From a clinical and policy perspective, type of admission, either planned versus unscheduled (admissions with less than 24 hours’ notice), cause of admission, and length of stay are important features of hospital readmissions. Frequency of unscheduled readmission is commonly used as a measure of quality of care and cost-efficiency as well as reflecting the health status of an individual,^6^ while length of stay is crucial for effective planning and management of hospital resources.^7^ However, there is still uncertainty around whether short length of stay/early discharge (which patients prefer) or longer length of hospital stay could be most beneficial to patients who are readmitted. Previous studies investigating hospital readmission following stroke have mainly assessed time to first all-cause and/or unscheduled readmission^8–11^ and have failed to account for total time spent as an unscheduled admission in hospital, which may be a more significant outcome for patients and their families in terms of symptom burden and quality of life.^12,13^ A clear understanding of the factors associated with unscheduled readmission is needed more than ever, particularly with healthcare systems being under an increasing amount of strain.

## Aims

The aims of this study were to:

- Assess unscheduled readmissions and time spent as an unscheduled admission in hospital during the first year after initial discharge from hospital following a stroke.
- Identify potential factors which could put patients at increased risk of unscheduled readmission thus enabling targeted support to reduce these.

## Methods

### Study design and data sources

This retrospective population-level data-linkage study used data obtained from Public Health Scotland (PHS), and National Records Scotland (NRS). The primary data source was the Scottish Stroke Care Audit (SSCA) which captures all acute stroke admissions to Scottish hospitals. For all patients in SSCA, data were extracted from Scottish Morbidity Record (SMR) 01 which contains nationally collated episode data for all hospitals, Prescribing Information System (PIS) which contains National Health Service (NHS) community prescribing data, and National Records of Scotland (NRS) death records including all deaths in Scotland. Detailed dataset information can be found in Supplementary Material. The STROBE-RECORD reporting guideline was followed^14^ (Supplementary Material).

### Data linkage

Pseudo-anonymised person-level linked SSCA, SMR01, PIS and NRS deaths dataset records were received from electronic Data Research and Innovation Service (eDRIS) of PHS, Scotland, UK. Records are linked using deterministic linkage via the community health index (CHI) number, a unique identifier for all residents in Scotland with a linkage rate of approximately 98%.^15^

### Study population

All patients (⩾18 years of age) admitted and subsequently discharged following first-ever stroke between July 1, 2010, to December 31, 2018, were included. SSCA data from 2005 and SMR 01 data from 1995 were used to exclude patients who had a previous International Classification of Diseases: 10th Revision (ICD-10) code for stroke.

### Outcome

#### Total time spent in hospital as unscheduled readmission in the year following discharge for stroke

Nationally collated hospital episode data, coded as unscheduled or planned admission, allowed for calculation of the burden of post-stroke unscheduled hospital readmission in the year following stroke discharge. Total time spent in hospital for unscheduled admissions in the first year following hospital discharge from first-ever stroke was defined as the number of distinct days spent in hospital coded as an unscheduled admission in relation to the number of days a patient was alive during the 1-year follow-up.

#### Patient and health-system factors

Patient and health-system factors in this study included: age, sex, modified 10-year Charlson Comorbidity Index (CCI) score (including conditions such as cancer, cardiac, respiratory disease, and diabetes), 2-year hospital frailty score (derived from prior hospital admissions where 0 = no frailty, <5 mild frailty, 5–14 moderate frailty, ⩾15 severe frailty), atrial fibrillation at time of stroke admission, hypertension, depression, pre-hospital prescribing, deprivation, urban-rurality classification, stroke severity, administration of a stroke care bundle, and thrombolysis. Information on how these factors were derived and categorized can be found in Supplementary Material and Supplemental Table S1.

### Statistical analysis

Statistical analyses were performed using SAS 9.4^®^ and R 4.4.2™. Descriptive statistics summarized the baseline characteristics for the study cohort at stroke admission. Categorical variables were summarized by frequencies (percentages) and compared using Pearson Chi-Square test. Continuous variables were summarized using the median (interquartile range (IQR)) and compared using Wilcoxon rank sum test. Since less than 0.2% of the study population had missing data, we excluded them from the analysis because such a small proportion is unlikely to introduce significant bias to our results.

Zero-inflated negative binomial regression models were used to explore the association between total time spent in hospital as unscheduled admission in the year following discharge in relation to potential risk factors. The log offset in the models was the number of days a patient was alive during the 1-year follow-up. A zero-inflated model takes account of data that have an incidence of zeros greater than expected for the underlying probability distribution.^16^ Description and analysis on time to first unscheduled readmission in relation to potential risk factors and unadjusted estimates for all analyses are included in Supplementary Material and Supplemental Tables S3–S5.

## Results

### Patient cohort

**Figure 1.**
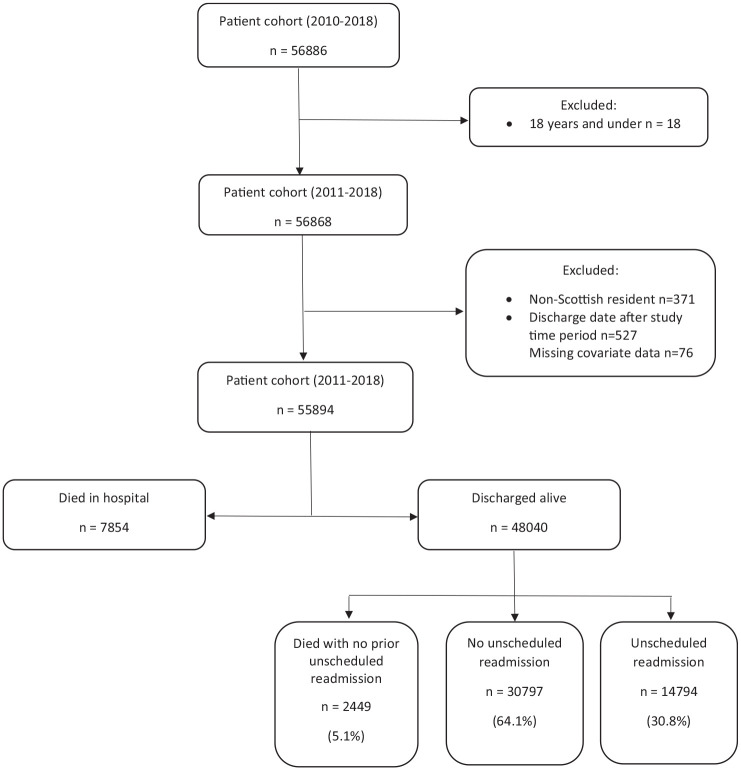
Study flow diagram.

The study cohort included 55,894 patients with first-ever stroke, 7854 died in hospital, resulting in a final study cohort of 48,040 patients who were discharged alive from hospital (Figure 1).

### Patient characteristics

Patient and care pathway characteristics for the study cohort at time of stroke admission and according to death in hospital or discharge from hospital are shown in Supplemental Table S2. Patient and care pathway characteristics for the final study cohort at stroke admission and according to unscheduled readmission categories are shown in Table 1. For all patients, median age was 73 years [IQR 63–82], 48.7% were female. Around 24.6% of patients lived in the most deprived areas, and 9.4% were classified as severe stroke. 42.5% had a pre-stroke CCI ⩾ 1 and 32.8% had pre-stroke frailty score ⩾ 1. For patients with unscheduled readmission, median age was 76 years [IQR 66–83], 51.5% female, 26.8% of patients lived in the most deprived areas, and 9.6% classified as severe stroke. Around 54.1% had a pre-stroke CCI ⩾ 1 and 44.7% had pre-stroke frailty score ⩾ 1.

**Table 1.** Patient and care pathway characteristics for unscheduled readmission.

	All	Unscheduled readmission (1-year)		
	N (%)	No n (%)	Yes n (%)	Died with no prior unscheduled readmission (competing event) n (%)
**n**	48,040	30,797	14,794	2449
Characteristic				
Age, years, at admission (median [interquartile range]	73 [63–82]	71 [61–80]	76 [66–83]	83 [77–89]
*Age group*				
<50 years	3268 (6.8)	2463 (8.0)	781 (5.3)	24 (1.0)
50–59 years	5877 (12.2)	4336 (14.1)	1471 (9.9)	70 (2.9)
60–69 years	9750 (20.3)	6943 (22.5)	2601 (17.6)	206 (8.4)
70–79 years	13,930 (29.0)	9011 (29.3)	4394 (29.6)	525 (21.4)
≥80 years	15,215 (31.7)	8044 (26.1)	5547 (37.5)	1624 (66.3)
Female	23,414 (48.7)	14,389 (46.7)	7612 (51.5)	1413 (57.7)
*Scottish Index of Multiple Deprivation (SIMD)*				
SIMD 1 (most deprived)	11,814 (24.6)	7307 (23.7)	3965 (26.8)	542 (22.1)
SIMD 2	10,782 (22.4)	6716 (21.8)	3548 (24.0)	518 (21.2)
SIMD 3	9609 (20.0)	6245 (20.3)	2856 (19.3)	508 (20.7)
SIMD 4	8251 (17.2)	5364 (17.4)	2396 (16.2)	491 (20.0)
SIMD 5 (least deprived)	7584 (15.8)	5165 (16.8)	2029 (13.7)	390 (15.9)
*Urban Rural Classification*				
Large urban areas	16,167 (33.7)	10,430 (33.9)	4927 (33.3)	810 (33.1)
Other urban areas	17,808 (37.1)	11,233 (36.5)	5666 (38.3)	909 (37.1)
Accessible small towns	4018 (8.4)	2572 (8.4)	1234 (8.3)	212 (8.7)
Remote small towns	1932 (4.0)	1214 (3.9)	620 (4.2)	98 (4.0)
Accessible rural areas	5175 (10.8)	3444 (11.2)	1485 (10.0)	246 (10.0)
Remote rural areas	2940 (6.1)	1904 (6.2)	862 (5.8)	174 (7.1)
Independent pre-stroke	41,787 (87.0)	28,019 (91.0)	12,344 (83.4)	1424 (58.1)
Living alone pre-stroke	18,154 (37.8)	11,002 (35.7)	6168 (41.7)	984 (40.2)
Can walk at admission	26,512 (55.2)	18,201 (59.1)	7604 (51.4)	707 (28.9)
Can talk at admission	39,234 (81.7)	25,829 (83.9)	11,879 (80.3)	1526 (62.3)
Orientated at admission	35,033 (72.9)	23,659 (76.8)	10,318 (69.7)	1056 (43.1)
Can lift both arms at admission	33,803 (70.4)	22,510 (73.1)	10,188 (68.9)	1105 (45.1)
*Stroke Severity*				
0 (least severe)	20,327 (42.3)	14,416 (46.8)	5509 (37.2)	402 (16.4)
1	10,387 (21.6)	6512 (21.1)	3470 (23.5)	405 (16.5)
2	9325 (19.4)	5587 (18.1)	3155 (21.3)	583 (23.8)
3	3463 (7.2)	1825 (5.9)	1233 (8.3)	405 (16.5)
4 (most severe)	4538 (9.4)	2457 (8.0)	1427 (9.6)	654 (26.7)
Hospital admission in 90 days pre-stroke admission	9110 (19.0)	4423 (14.4)	3930 (26.6)	757 (30.9)
Prior TIA	1905 (4.0)	1055 (3.4)	739 (5.0)	111 (4.5)
In-hospital stroke	1796 (3.7)	727 (2.4)	842 (5.7)	227 (9.3)
Intracerebral hemorrhage	4508 (9.4)	2872 (9.3)	1350 (9.1)	286 (11.7)
*Pre-stroke Charlson Comorbidity Index (CCI)*				
0	27,611 (57.5)	19,966 (64.8)	6791 (45.9)	854 (34.9)
1	7750 (16.1)	4620 (15.0)	2638 (17.8)	492 (20.1)
2	5951 (12.4)	3301 (10.7)	2228 (15.1)	422 (17.2)
≥ 3	6728 (14.0)	2910 (9.4)	3137 (21.2)	682 (27.8)
*Pre-stroke Charlson comorbidity count*				
0	27,611 (57.5)	19,966 (64.8)	6791 (45.9)	854 (34.9)
1	11,134 (23.2)	6586 (21.4)	3815 (25.8)	733 (29.9)
2	5477 (11.4)	2750 (8.9)	2260 (15.3)	467 (19.1)
≥ 3	3818 (7.9)	1495 (4.9)	1928 (13.0)	395 (16.1)
*Pre-stroke comorbidities*				
Myocardial infarction	4505 (9.4)	2240 (7.3)	1928 (13.0)	337 (13.8)
Congestive heart failure	3176 (6.6)	1423 (4.6)	1415 (9.6)	338 (13.8)
Cancer	4836 (10.1)	2504 (8.1)	1892 (12.8)	440 (18.0)
Renal disease	3533 (7.4)	1523 (4.9)	1642 (11.1)	368 (15.0)
Liver disease	790 (1.6)	388 (1.3)	365 (2.5)	37 (1.5)
Diabetes mellitus	8432 (17.6)	4683 (15.2)	3264 (22.1)	485 (19.8)
Peripheral vascular disease	2560 (5.3)	1208 (3.9)	1163 (7.9)	189 (7.7)
Atrial fibrillation diagnosed at time of stroke admission	9892 (20.6)	5547 (18.0)	3513 (23.7)	832 (34.0)
Hypertension	31,357 (65.3)	18,747 (60.9)	10,727 (72.5)	1833 (76.9)
Depression	10,998 (22.9)	6348 (20.6)	3993 (27.0)	657 (26.8)
*Pre-stroke frailty index category*				
None	32,298 (67.2)	22,983 (74.6)	8176 (55.3)	1139 (46.5)
Mild	9257 (19.3)	5096 (16.5)	3634 (24.6)	527 (21.5)
Moderate	5297 (11.0)	2342 (7.6)	2387 (16.1)	568 (23.2)
Severe	1188 (2.5)	376 (1.2)	597 (4.0)	215 (8.8)
*Medication prior to stroke admission*				
Antihypertensives	29,701 (61.8)	17,909 (58.2)	10,084 (68.2)	1708 (69.7)
Statins	20,281 (42.2)	12,229 (39.7)	7007 (47.4)	1045 (42.7)
Antiplatelets	17,140 (35.7)	9791 (31.8)	6264 (42.3)	1085 (44.3)
Anticoagulants	4015 (8.4)	2089 (6.8)	1592 (10.8)	334 (13.6)
Initial stroke admission length of hospital stay (median [interquartile range])	9 [3–34]	7 [3–25]	12 [4–44]	29 [9–77]
Discharged to usual residence	38,558 (80.3)	25,610 (83.2)	11,741 (79.4)	1207 (49.3)
*Stroke care bundle components*				
*All patients*				
Stroke unit	36,310 (75.6)	23,386 (75.9)	11,152 (75.4)	1772 (72.4)
Brain scan	43,908 (91.4)	28,365 (92.1)	13,354 (90.3)	2189 (89.4)
Swallow screen	43,066 (89.6)	27,729 (90.0)	13,142 (88.8)	2195 (89.6)
*Ischaemic stroke only*	43,532	27,925	13,444	2163
Early antiplatelet treatment	37,893 (87.0)	24,385 (87.3)	11,637 (86.6)	1871 (86.5)
Complete stroke care bundle	31,184 (64.9)	20,142 (65.4)	9502 (64.2)	1540 (62.9)
*Ischaemic stroke only*	43,532	27,925	13,444	2163
Intravenous thrombolysis	3880 (8.9)	2435 (8.7)	1262 (9.4)	183 (8.5)

### Unscheduled readmissions over the follow-up period

Table 2 shows the frequency of patients first unscheduled readmission at specific time periods during 1-year follow-up. During this period, 14,794 (30.8%) had at least one unscheduled readmission, 2449 (5.1%) died with no prior readmission, and 30,797 (64.1%) survived with no unscheduled readmission (Table 2).

**Table 2.** Frequency of patient first unscheduled readmission according to time following stroke discharge.

	1–30 days n (%)	31–90 days n (%)	91–365 days n (%)	Total (n = 48,040)
Unscheduled readmission	3821 (25.8)	3451 (23.3)	7522 (50.9)	14,794
Died with no prior unscheduled readmission (competing event)	813 (33.2)	611 (24.9)	1025 (41.9)	2449

The number of unscheduled readmissions per patient and median time spent in hospital during 1-year follow-up post-stroke discharge is shown in Table 3. Of the patients who had an unscheduled readmission, 37.2% had more than one unscheduled readmission during the 1-year follow-up. The median time spent in hospital as an unscheduled readmission in the 1-year follow-up was 9 days [IQR 3–25].

**Table 3.** Number of unscheduled readmissions per patient and median time spent in hospital during 1-year follow-up post-stroke discharge.

Number of unscheduled readmissions	n (%) (total patients = 14,794)
1	9285 (62.8)
2	3229 (21.8)
3	1245 (8.4)
≥ 4	1035 (7.0)
Time spent in hospital (days) (median [interquartile range])	9 [3–25]

### Total time spent in hospital as an unscheduled readmission in the year following discharge for stroke

**Figure 2.**
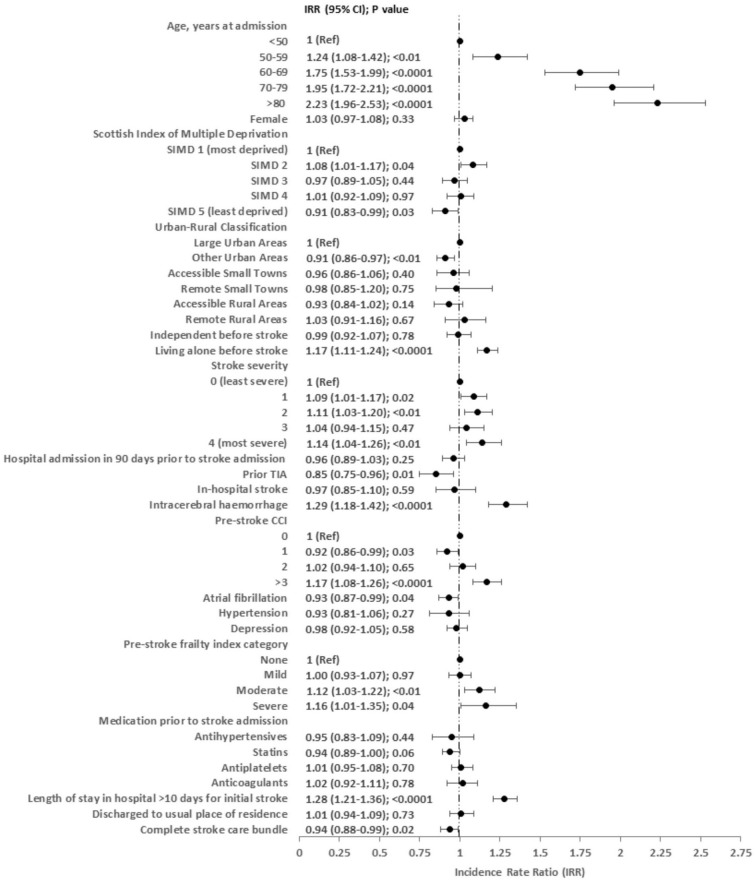
Multivariable zero-inflated negative binomial estimations for number of days spent in hospital as an unscheduled admission during 1-year follow-up.

After adjustment, an increased risk of unscheduled hospital readmission time was associated with increasing age (e.g. ≥ 80 years versus <50 years IRR 2.23 (95% CI 1.96–2.53)); living alone before stroke (IRR 1.17 (95% CI 1.11–1.24)); stroke severity (most versus least severe IRR 1.14 (95% CI 1.04–1.26)); intracerebral hemorrhage (IRR 1.29 (95% CI 1.18–1.42)); higher CCI (⩾3 versus 0 IRR 1.17 (95% CI 1.08–1.26)); higher frailty index (severe versus none IRR 1.16 (95% CI 1.01–1.35), moderate versus none IRR 1.12 (95% CI 1.03–1.22)); and longer length of stay for initial stroke admission (>10 days IRR 1.28 (95% CI 1.21–1.36)) (Figure 2).

A decreased risk of patient secondary care unscheduled readmission time was associated with prior TIA (IRR 0.85 (95% CI (0.75–0.96)); and a weak association with lower socio-economic deprivation (least versus most deprived IRR 0.91 (95% CI 0.83–0.99)); and receipt of complete stroke care bundle (IRR 0.94 (95% CI 0.88–0.99)) (Figure 2).

## Discussion

### Summary of main results

To the best of our knowledge this is the first study exploring how patient and health-system factors impact unscheduled hospital readmission time within 1-year of stroke discharge. In this national cohort, over one-third had at least one unscheduled readmission within 1-year post-stroke discharge. Patient and health-system factors associated with increased unscheduled hospital readmission time included increasing age, living alone pre-stroke, higher comorbidity burden, higher frailty index, more severe strokes, intracerebral hemorrhage, and length of stay for initial stroke admission greater than 10 days. Conversely, lower socio-economic deprivation, prior TIA, and receipt of a complete stroke care bundle were associated with reduced unscheduled hospital readmission time in the 1-year post-stroke discharge.

### Context with other studies

In terms of unscheduled readmission rates within 1 year of stroke discharge (30.8%) and supplementary results on factors associated with time to first unscheduled readmission, our findings align with prior studies.^5,9^ Although various factors have been previously identified as being associated with hospital readmission following stroke, including age, prior stroke, prolonged length of stay, comorbidity, and frailty,^9,17–19^ most studies have included planned/elective admissions, which are more commonly associated with non-urgent diagnoses or follow-up care.^20^ In comparison to previous studies, our study has not only considered unscheduled readmissions, which occur unexpectedly often necessitating urgent medical attention, but also incorporated time spent in hospital as an unscheduled readmission over the course of 1-year following discharge for stroke, reflecting the economic burden of such admissions.

A significant finding was that increased time spent in hospital in these circumstances was predominantly related to patient-level factors present before stroke admission rather than health-system factors. This is consistent with other conditions where clinical processes of care had only very weak, or no relationship to the risk of readmission.^21^ Factors including increasing age, deprivation, comorbidity, and previous health-care use have been shown to be stronger predictors of hospital resource use than factors associated with the acute illness itself.^22^ Prevention of unscheduled readmission may therefore require greater standardization of interventions focused during the pre- and post-discharge period, with individualisation where appropriate. While further research to assess the impact of early supported discharge on unscheduled readmissions is needed, we suggest all discharge plans should include optimal management of factors which might predispose to unscheduled readmission.

Living alone pre-stroke increased the time spent in hospital as an unscheduled admission, in line with previous studies investigating other conditions which demonstrate an association with increased risk.^23–25^ Following stroke, individuals living at home with home care services were shown to be at highest risk of readmission.^26^ Living alone is a risk factor of social isolation and may also have a detrimental effect on a person’s mobility, nutrition, and medication compliance.^27,28^ Patients living alone may also have limited access to information, health, and community support services, which may contribute to adverse patient outcomes. It is important to acknowledge that not all individuals living alone are socially isolated or have no formal or informal care assistance, and we were unable to differentiate using the routine administrative data available in this study. However, even after correcting for factors associated with living alone, including prior hospital use, independence pre-stroke, and frailty, living alone was still significant. This suggests there is an additional healthcare and support need in terms of considering living circumstances for discharge and care planning, targeting individuals at risk of unscheduled readmission with community-based pre-emptive interventions is therefore important after stroke, as readmissions may be potentially avoidable.

Higher pre-stroke comorbidity burden was associated with increased time spent as an unscheduled readmission 1-year post-stroke discharge, consistent with previous studies,^9^ and moderate to severe pre-stroke frailty, resulting from the cumulative decline in many physiological systems, also showed an increased association. Pre-stroke frailty has been previously shown to be independently associated with stroke severity in the acute setting^29^ and associated with marked reduction in self-reported quality of life following stroke.^30^ However, higher percentages of pre-morbidly frail patients have also been shown to be discharged to a care home setting following stroke admission compared to non-frail patients (46.9% vs 18.5%),^31^ which could potentially help mitigate against increased unscheduled readmission for reasons including advanced directives or regular primary care input. Increasing stroke severity was associated with increased time spent as an unscheduled readmission, and this may reflect reduced independence following stroke and perhaps a lower threshold for admission which we have not been able to measure. Prior TIA was associated with decreased time spent as an unscheduled readmission which might reflect unmeasured factors such as health awareness and lifestyle.

Receipt of a stroke care bundle has previously been shown to reduce the risk of dying and increase discharge to usual place of residence.^32^ All Scottish health boards are held to the same standards for stroke care in implementing the stroke care bundle, with annual review highlighting areas for improvement. Receipt of the complete stroke care bundle reduced the time spent in hospital as an unscheduled readmission suggesting this is an important component of stroke care and although resource allocation pressures in hospital may make this difficult to achieve for all patients, access to appropriate acute stroke care from stroke-trained staff is important in reducing unscheduled readmission post-stroke.

### Strengths and limitations

The data in this study were derived from comprehensive and high-quality clinical datasets, including the SSCA which accurately represents stroke care in this population,^33^ which can be linked through a patient’s unique healthcare CHI number.^15^ Compared to previous studies, hospital inpatient admission data enabled greater exploration of post-stroke unscheduled readmissions over the course of 1-year post-discharge providing a more patient-centered outcome than time to first readmission, as total time spent as an unscheduled readmission may be a more significant outcome for patients and their families in terms of symptom burden and quality of life. Universal NHS care in Scotland means our findings are not confounded by personal liability for healthcare and medication costs.

A limitation is that we are restricted to reporting on outcome measures and adjusting for confounders which were available in the clinical datasets. Unmeasured variables such as ease of access to rehabilitation therapists, supported discharge teams, or withdrawal of care and their variability between hospitals may have influenced differences in unscheduled readmission. Secondary care also represents only one dimension of healthcare so our study does not reflect the whole spectrum of health and social care which could influence post-diagnosis secondary unscheduled readmission time, including the extent to which patients may have chosen to accept further treatments or accessed palliative care.

## Conclusion

Overall, our results suggest there are several factors associated with time spent as an unscheduled hospital readmission following stroke discharge. With an aging population and healthcare systems under increasing amount of pressure, efficient management strategies, resource allocations, and community support are crucial. A holistic pre-discharge assessment with interventions to minimize readmission risks associated with living arrangements and frailty, and medical assessment to optimize management and secondary prevention of comorbidities could benefit patients and the healthcare system.

## Supplemental Material

sj-docx-1-wso-10.1177_17474930251355864 – Supplemental material for Factors influencing time spent in hospital for unscheduled readmissions after stroke discharge Supplemental material, sj-docx-1-wso-10.1177_17474930251355864 for Factors influencing time spent in hospital for unscheduled readmissions after stroke discharge by Melanie Turner, David J McLernon, Peter Murchie, Peter Langhorne and Mary-Joan Macleod in International Journal of Stroke
