# Supplementary material for: Factors influencing time spent in hospital for unscheduled readmissions after stroke discharge
Source: Int J Stroke. 2025 Jun 25;20(10):1290–300. doi: 10.1177/17474930251355864 (PMC12664915; doi:10.1177/17474930251355864)
Supplement: sj-docx-1-wso-10.1177_17474930251355864 – Supplemental material for Factors influencing time spent in hospital for unscheduled readmissions after stroke discharge [file sj-docx-1-wso-10.1177_17474930251355864.docx]

**Supplementary**

*Supplemental Methods*

*Dataset Information*

Scottish Stroke Care Audit (SSCA) is a national audit program that monitors the quality of stroke care provided in hospitals across Scotland. SSCA was established in 2002 to monitor performance against guideline-based Clinical Standards. It provides detailed information on strokes admitted to all Scottish hospitals: baseline covariates (including demographics, brain imaging, stroke classification, stroke prevention medication, presence of atrial fibrillation) and outcomes (including achievement of a stroke care bundle, length of stay in hospital, discharge destination).

Scottish Morbidity Record 01 (SMR01) collects episode level data on hospital inpatient and day case discharges from hospitals in Scotland. The dataset contains detailed information on admission date, admission type, ICD-10 condition codes, operations, discharge location, Scottish Index of Multiple Deprivation, and urban/rurality codes.

Prescribing Information System (PIS) contains data for prescribing relating to all medicines that are prescribed and dispensed in the community in Scotland, as well as prescriptions written in hospital that are dispensed in the community. Data includes prescribed date, dispensed date, approved drug name, prescribable item name, drug item formulation, drug item strength, and BNF item code.

National Records of Scotland (NRS) is the official source for all Scottish death records and includes information on date of death, and cause(s) of death.

*Patient and health-system factors*

Comorbid conditions and a weighted-modified 10-year Charlson Comorbidity Index (CCI) score (excluding cerebrovascular disease and paraplegia due to their correlation with stroke) (1), and a weighted two-year frailty score (2) were derived from principal and supplementary diagnostic ICD-10-CM codes based on nationally collated hospital episode data from SMR01 (3).

The International Classification of Diseases, Tenth Revision, Clinical Modification codes used to calculate the modified Charlson Comorbidity Index (CCI) score are as follows:

*Myocardial Infarction*: I21, I22, I25.2

*Congestive heart failure*: I09.9, I11.0, I13.0, I13.2, I25.5, I42.0, I42.5–I42.9, I43, I50, P29.0

*Peripheral vascular disease*: I70, I71, I73.1, I73.8-I73.9, I77.1, I79.0, I79.2, K55.1, K55.8-K55.9, Z95.8-Z95.9

*Dementia*: F00–F03, F05.1, G30, G31.1

*Chronic pulmonary disease*: I27.8, I27.9, J40- J47, J60–J67, J68.4, J70.1, J70.3

*Rheumatologic disease*: M05, M06, M31.5, M32–M34, M35.1, M35.3, M36.0

*Peptic ulcer*: K25–K28

*Mild liver disease*: B18, K70.0–K70.3, K70.9, K71.3–K71.5, K71.7, K73, K74, K76.0, K76.2–K76.4, K76.8, K76.9, Z94.4

*Diabetes without chronic complications*: E10.0–E10.1, E10.6, E10.8-E10.9, E11.0-E11.1, E11.6, E11.8-E11.9, E12.0-E12.1, E12.6, E12.8-E12.9, E13.0-E13.1, E13.6, E13.8-E13.9, E14.0-E14.1, E1.6, E14.8-E14.9

*Diabetes with chronic complications*: E10.2–E10.5, E10.7, E11.2–E11.5, E11.7, E12.2–E12.5, E12.7, E13.2–E13.5, E13.7, E14.2–E14.5, E14.7

*Renal disease*: I12.0, I13.1, N03.2–N03.7, N05.2– N05.7, N18, N19, N25.0, Z49.0– Z49.2, Z94.0, Z99.2

*Any malignancy, including leukaemia and lymphoma*: C00–C26, C30–C34, C37–C41, C43, C45–C58, C60–C76, C81–C85, C88, C90–C97

*Moderate or severe liver disease*: I85.0, I85.9, I86.4, I98.2, K70.4, K71.1, K72.1, K72.9, K76.5–K76.7

*Metastatic solid tumour*: C77–C80

*AIDS/HIV*: B20–B22, B24

Pre-hospital prescribing was derived from the Prescribing Information System (PIS) dataset of all community dispensed NHS prescriptions. Using residential postcodes, the Scottish Index of Multiple Deprivation (SIMD), an area-based measure of deprivation, was assigned to the data (4) along with the Scottish Government six-fold urban-rural classification (5). NRS death records were used to determine date of death and all-cause mortality (6). Patient follow-up was until 31^st^ December 2019.

SSCA does not collect National Institutes of Health Stroke Scale (NIHSS) but does collect information based on the six simple variables (SSV) model which has previously been shown to predict the outcome of being alive and independent at six months and one year after stroke (7). Stroke severity was calculated as a composite scale based on a patient’s ability to walk, talk, lift both arms, and orientate to person, time, place, derived from the six simple variables (SSV) model. The overall score was calculated from ‘0 to 4’; 0 indicates least severe and 4 most severe. A score of 4 represents a patient unable to perform any of these (classed as most severe stroke). Unpublished analysis from our research group using a subset of SSCA patients with NIHSS values shows that NIHSS is highly correlated with stroke severity using the composite scale based on a patient’s ability to walk, talk, lift both arms, and orientate to person, time and place.

The SSCA monitors the quality of care provided by Scottish hospitals using agreed standards: admission to a stroke unit within one day of presentation, brain imaging within 12 hours of admission, swallow screen assessment within four hours of admission and for ischaemic stroke patients, administration of aspirin (unless contraindicated) within one day of admission. Standards for brain imaging and swallow screen were refined in 2015 and implemented in 2016, as described in Supplementary Table 1. Achievement of the complete bundle was defined as receiving the three components for haemorrhagic and four components for ischaemic stroke. Thrombolysis administration was also recorded.

In addition to comorbidities in the CCI, the comorbidities of atrial fibrillation and hypertension were also recorded due to their association with stroke. Diagnosis of atrial fibrillation at time of stroke admission is recorded in the SSCA and a diagnosis of hypertension was derived from principal and supplementary diagnostic ICD-10-CM codes based on nationally collated hospital episode data (SMR01) and/or a prescription for any anti-hypertensive medication in the six months prior to stroke admission. Diagnosis of depression was derived from principal and supplementary diagnostic ICD-10-CM codes based on nationally collated hospital episode data (SMR01) and/or a prescription for any anti-depressant medication in the six months prior to stroke admission. To determine medication use prior to stroke admission, pre-hospital prescribing in the six months prior to hospital admission was calculated from prescribing data from the Prescribing Information Systems (PIS) dataset.

Table S1. Stroke care standard definitions

| **Stroke care bundle component** | **Definition prior to 2015** | **Definition 2015 onwards** |
| --- | --- | --- |
| Stroke unit admission | Within one day of admission | Within one day of admission |
| Brain imaging | Within 24 hours of admission | Within 12 hours of admission |
| Swallow screen assessment | Within one day of admission | Within four hours of admission |
| Administration of aspirin | Within one day of admission | Within one day of admission |

*Time to first unscheduled readmission*

Nationally collated hospital episode data allowed for calculation of time to first unscheduled hospital readmission in the first year following hospital discharge after a stroke. This was defined as the number of distinct days from date of discharge for index stroke event to date of first unscheduled readmission. Competing risk analysis using a Fine and Gray sub-distribution hazards model, with death without unscheduled readmission being the competing risk, was carried out to explore time to unscheduled readmission in relation to potential risk factors. Proportional hazards assumptions were checked.

Table S2. Patient characteristics for study cohort admitted to hospital with stroke

|  | **All**  **N (%)** | **Died in hospital**  **n (%)** | **Discharged alive**  **n (%)** | **P-value** |
| --- | --- | --- | --- | --- |
| **n** | 55894 | 7854 | 48040 |  |
| **Characteristic** |  |  |  |  |
| Age, years, at admission (median [interquartile range] | 75 [64-83] | 82 [75-88] | 73 [63-82] | <0.0001 |
|  |  |  |  |  |
| *Age group* |  |  |  |  |
| <50 years | 3382 (6.1) | 114 (1.5) | 3268 (6.8) | <0.001 |
| 50-59 years | 6146 (11.0) | 269 (3.4) | 5877 (12.2) |  |
| 60-69 years | 10478 (18.7) | 728 (9.3) | 9750 (20.3) |  |
| 70-79 years | 15864 (28.4) | 1934 (24.6) | 13930 (29.0) |  |
| >80 years | 20024 (35.8) | 4809 (61.2) | 15215 (31.7) |  |
|  |  |  |  |  |
| Women | 27958 (50.0) | 4544 (57.9) | 23414 (48.7) | <0.001 |
|  |  |  |  |  |
| *Scottish Index of Multiple Deprivation (SIMD)* |  |  |  |  |
| SIMD 1 (most deprived) | 13508 (24.2) | 1694 (21.6) | 11814 (24.6) | <0.001 |
| SIMD 2 | 12612 (22.6) | 1830 (23.3) | 10782 (22.4) |  |
| SIMD 3 | 11256 (20.1) | 1647 (21.0) | 9609 (20.0) |  |
| SIMD 4 | 9677 (17.3) | 1426 (18.2) | 8251 (17.2) |  |
| SIMD 5 (least deprived) | 8841 (15.8) | 1257 (16.0) | 7584 (15.8) |  |
|  |  |  |  |  |
| *Urban Rural Classification* |  |  |  |  |
| Large Urban Areas | 18755 (33.6) | 2588 (33.0) | 16167 (33.7) | 0.748 |
| Other Urban Areas | 20714 (37.1) | 2906 (37.0) | 17808 (37.1) |  |
| Accessible Small Towns | 4694 (8.4) | 676 (8.6) | 4018 (8.4) |  |
| Remote Small Towns | 2261 (4.0) | 329 (4.2) | 1932 (4.0) |  |
| Accessible Rural Areas | 6030 (10.8) | 855 (10.9) | 5175 (10.8) |  |
| Remote Rural Areas | 3440 (6.2) | 500 (6.4) | 2940 (6.1) |  |
|  |  |  |  |  |
| Independent pre-stroke | 47056 (84.2) | 5269 (67.1) | 41787 (87.0) | <0.001 |
| Living alone pre-stroke | 21589 (38.6) | 3435 (43.7) | 18154 (37.8) | <0.001 |
| Can walk at admission | 27595 (49.4) | 1083 (13.8) | 26512 (55.2) | <0.001 |
| Can talk at admission | 42319 (75.7) | 3085 (39.3) | 39234 (81.7) | <0.001 |
| Orientated at admission | 37163 (66.5) | 2130 (27.1) | 35033 (72.9) | <0.001 |
| Can lift both arms at admission | 35581 (63.7) | 1778 (22.6) | 33803 (70.4) | <0.001 |
| *Stroke Severity* |  |  |  |  |
| 0 (least severe) | 20842 (37.3) | 515 (6.6) | 20327 (42.3) | <0.001 |
| 1 | 11041 (19.8) | 654 (8.3) | 10387 (21.6) |  |
| 2 | 10771 (19.3) | 1446 (18.4) | 9325 (19.4) |  |
| 3 | 4625 (8.3) | 1162 (14.8) | 3463 (7.2) |  |
| 4 (most severe) | 8615 (15.4) | 4077 (51.9) | 4538 (9.4) |  |
|  |  |  |  |  |
| Hospital admission in 90 days pre-stroke admission | 11668 (20.9) | 2558 (32.6) | 9110 (19.0) | <0.001 |
| Prior TIA | 2247 (4.0) | 342 (4.4) | 1905 (4.0) | 0.104 |
|  |  |  |  |  |
| In-hospital stroke | 2697 (4.8) | 901 (11.5) | 1796 (3.7) | <0.001 |
| Intracerebral haemorrhage | 6371 (11.4) | 1863 (23.7) | 4508 (9.4) | <0.001 |
|  |  |  |  |  |
| *Pre-stroke Charlson Comorbidity Index (CCI)* |  |  |  |  |
| 0 | 30681 (54.9) | 3070 (39.1) | 27611 (57.5) | <0.001 |
| 1 | 9113 (16.3) | 1363 (17.4) | 7750 (16.1) |  |
| 2 | 7216 (12.9) | 1265 (16.1) | 5951 (12.4) |  |
| >3 | 8884 (15.9) | 2156 (27.5) | 6728 (14.0) |  |
|  |  |  |  |  |
| *Pre-stroke Charlson comorbidity count* |  |  |  |  |
| 0 | 30681 (54.9) | 3070 (39.1) | 27611 (57.5) | <0.001 |
| 1 | 13278 (23.8) | 2144 (27.3) | 11134 (23.2) |  |
| 2 | 6862 (12.3) | 1385 (17.6) | 5477 (11.4) |  |
| >3 | 5073 (9.1) | 1255 (16.0) | 3818 (7.9) |  |
|  |  |  |  |  |
| *Pre-stroke comorbidities* |  |  |  |  |
| Myocardial Infarction | 5612 (10.0) | 1107 (14.1) | 4505 (9.4) | <0.001 |
| Congestive heart failure | 4314 (7.7) | 1138 (14.5) | 3176 (6.6) | <0.001 |
| Cancer | 6170 (11.0) | 1334 (17.0) | 4836 (10.1) | <0.001 |
| Renal disease | 4702 (8.4) | 1169 (14.9) | 3533 (7.4) | <0.001 |
| Liver disease | 955 (1.7) | 165 (2.1) | 790 (1.6) | 0.004 |
| Diabetes Mellitus | 9923 (17.8) | 1491 (19.0) | 8432 (17.6) | 0.002 |
| Peripheral vascular disease | 3244 (5.8) | 684 (8.7) | 2560 (5.3) | <0.001 |
|  |  |  |  |  |
| Atrial fibrillation diagnosed at time of stroke admission | 12788 (22.9) | 2896 (36.9) | 9892 (20.6) | <0.001 |
| Hypertension | 37515 (67.1) | 6158 (78.4) | 31357 (65.3) | <0.001 |
| Depression | 12832 (23.0) | 1834 (23.4) | 10998 (22.9) | 0.371 |
|  |  |  |  |  |
| *Pre-stroke frailty index category* |  |  |  |  |
| None | 36052 (64.5) | 3754 (47.8) | 32298 (67.2) | <0.001 |
| Mild | 11069 (19.8) | 1812 (23.1) | 9257 (19.3) |  |
| Moderate | 7068 (12.6) | 1771 (22.5) | 5297 (11.0) |  |
| Severe | 1705 (3.1) | 517 (6.6) | 1188 (2.5) |  |
|  |  |  |  |  |
| *Medication prior to stroke admission* |  |  |  |  |
| Antihypertensives | 35458 (63.4) | 5757 (73.3) | 29701 (61.8) | <0.001 |
| Statins | 24240 (43.4) | 3959 (50.4) | 20281 (42.2) | <0.001 |
| Antiplatelets | 20620 (36.9) | 3480 (44.3) | 17140 (35.7) | <0.001 |
| Anticoagulants | 5221 (9.3) | 1206 (15.4) | 4015 (8.4) | <0.001 |
|  |  |  |  |  |
| Initial stroke admission length of hospital stay (median [interquartile range]) | 9 [4-34] | 11 [4-31] | 9 [3-34] | <0.001 |
|  |  |  |  |  |
| *Stroke care bundle components* |  |  |  |  |
| *All patients* |  |  |  |  |
| Stroke unit | 41764 (74.7) | 5454 (69.4) | 36310 (75.6) | <0.001 |
| Brain scan | 51091 (91.4) | 7183 (91.5) | 43908 (91.4) | 0.866 |
| Swallow screen | 50242 (89.9) | 7176 (91.4) | 43066 (89.6) | <0.001 |
| *Ischaemic stroke only* | 49523 | 5991 | 43532 |  |
| Antiplatelet | 43154 (87.1) | 5261 (87.8) | 37893 (87.0) | 0.096 |
|  |  |  |  |  |
| Complete stroke care bundle | 35979 (64.4) | 4795 (61.1) | 31184 (64.9) | <0.001 |
|  |  |  |  |  |
| *Ischaemic stroke only* | 49523 | 5991 | 43532 |  |
| Intravenous thrombolysis | 4744 (9.6) | 864 (14.4) | 3880 (8.9) | <0.001 |

Table S3. Unadjusted zero-inflated negative binomial estimations for number of days spent in hospital as an unscheduled readmission during one-year follow-up

|  | Unadjusted IRR  (95% CI) | P value |
| --- | --- | --- |
|  |  |  |
| Age, years at admission |  |  |
| <50 | 1.00 (Ref) |  |
| 50-59 | 1.43 (1.25-1.64) | <0.0001 |
| 60-69 | 1.92 (1.70-2.18) | <0.0001 |
| 70-79 | 2.39 (2.12-2.69) | <0.0001 |
| >80 | 2.87 (2.56-3.23) | <0.0001 |
|  |  |  |
| Female | 1.15 (1.09-1.21) | <0.0001 |
|  |  |  |
| *Scottish Index of Multiple Deprivation (SIMD)* |  |  |
| SIMD 1 (most deprived) | 1.00 (Ref) |  |
| SIMD 2 | 1.09 (1.01-1.17) | 0.0229 |
| SIMD 3 | 1.05 (0.97-1.14) | 0.2169 |
| SIMD 4 | 1.02 (0.95-1.11) | 0.5418 |
| SIMD 5 (least deprived) | 0.93 (0.85-1.01) | 0.0905 |
|  |  |  |
| *Urban-Rural classification* |  |  |
| Large urban areas | 1.00 (Ref) |  |
| Other urban areas | 0.92 (0.87-0.98) | 0.0134 |
| Accessible small towns | 0.92 (0.83-1.02) | 0.125 |
| Remote small towns | 1.05 (0.92-1.20) | 0.4819 |
| Accessible rural areas | 0.91 (0.83-1.00) | 0.0515 |
| Remote rural areas | 0.99 (0.88-1.11) | 0.8614 |
|  |  |  |
| Independent before stroke | 0.69 (0.64-0.74) | <0.0001 |
| Living alone before stroke | 1.38 (1.31-1.45) | <0.0001 |
|  |  |  |
| *Stroke severity* |  |  |
| 0 (least severe) | 1.00 (Ref) |  |
| 1 | 1.32 (1.24-1.42) | <0.0001 |
| 2 | 1.40 (1.30-1.50) | <0.0001 |
| 3 | 1.31 (1.19-1.45) | <0.0001 |
| 4 (most severe) | 1.36 (1.24-1.49) | <0.0001 |
|  |  |  |
| Hospital admission in 90 days prior to stroke admission | 1.30 (1.23-1.38) | <0.0001 |
| Prior TIA | 1.01 (0.89-1.14) | 0.8929 |
| In-hospital stroke | 1.48 (1.32-1.65) | <0.0001 |
| Intracerebral haemorrhage | 1.18 (1.08-1.30) | 0.0002 |
|  |  |  |
| *Pre-stroke Charlson Comorbidity Index (CCI)* |  |  |
| 0 | 1.00 (Ref) |  |
| 1 | 1.11 (1.03-1.19) | 0.0063 |
| 2 | 1.28 (1.19-1.39) | <0.0001 |
| >3 | 1.57 (1.46-1.68) | <0.0001 |
|  |  |  |
| Atrial fibrillation diagnosed at time of stroke admission | 1.18 (1.11-1.26) | <0.0001 |
| Hypertension | 1.31 (1.24-1.39) | <0.0001 |
| Depression | 1.03 (0.98-1.10) | 0.2378 |
|  |  |  |
| *Pre-stroke frailty index category* |  |  |
| None | 1.00 (Ref) |  |
| Mild | 1.26 (1.15-1.30) | <0.0001 |
| Moderate | 1.60 (1.49-1.72) | <0.0001 |
| Severe | 1.95 (1.71-2.23) | <0.0001 |
|  |  |  |
| *Medication prior to stroke admission* |  |  |
| Antihypertensives | 1.24 (1.18-1.32) | <0.0001 |
| Statins | 1.12 (1.07-1.18) | <0.0001 |
| Antiplatelets | 1.19 (1.13-1.25) | <0.0001 |
| Anticoagulants | 1.18 (1.09-1.29) | <0.0001 |
|  |  |  |
| Length of stay in hospital >10 days for initial stroke | 1.52 (1.45-1.60) | <0.0001 |
|  |  |  |
| Discharged to usual place of residence | 0.79 (0.74-0.85) | <0.0001 |
|  |  |  |
| Complete stroke care bundle | 0.88 (0.84-0.93) | <0.0001 |

Table S4. Unadjusted competing risk analysis for unscheduled readmission during one-year follow-up

|  | Unadjusted HR  (95% CI) | P-value |
| --- | --- | --- |
| Age, years at admission |  |  |
| <50 | 1.00 (Ref) |  |
| 50-59 | 1.06 (0.97-1.15) | 0.2198 |
| 60-69 | 1.14 (1.05-1.23) | 0.0015 |
| 70-79 | 1.39 (1.28-1.49) | <0.0001 |
| >80 | 1.65 (1.53-1.78) | <0.0001 |
|  |  |  |
| Female | 1.14 (1.11-1.18) | <0.0001 |
|  |  |  |
| *Scottish Index of Multiple Deprivation (SIMD)* |  |  |
| SIMD 1 (most deprived) | 1.00 (Ref) |  |
| SIMD2 | 0.98 (0.93-1.02) | 0.2707 |
| SIMD 3 | 0.86 (0.82-0.91) | <0.0001 |
| SIMD 4 | 0.84 (0.80-0.89) | <0.0001 |
| SIMD 5 (least deprived) | 0.76 (0.72-0.81) | <0.0001 |
|  |  |  |
| *Urban-Rural classification* |  |  |
| Large Urban Areas | 1.00 (Ref) |  |
| Other Urban Areas | 1.05 (1.01-1.09) | 0.0086 |
| Accessible Small Towns | 1.01 (0.95-1.07) | 0.7851 |
| Remote Small Towns | 1.07 (0.99-1.17) | 0.1041 |
| Accessible Rural Areas | 0.93 (0.88-0.99) | 0.0131 |
| Remote Rural Areas | 0.95 (0.89-1.03) | 0.2030 |
|  |  |  |
| Independent pre-stroke | 0.70 (0.67-0.73) | <0.0001 |
| Living alone pre-stroke | 1.21 (1.18-1.25) | <0.0001 |
|  |  |  |
| *Stroke severity* |  |  |
| 0 (least severe) | 1.00 (Ref) |  |
| 1 | 1.28 (1.23-1.34) | <0.0001 |
| 2 | 1.30 (1.25-1.36) | <0.0001 |
| 3 | 1.40 (1.31-1.48) | <0.0001 |
| 4 (most severe) | 1.20 (1.14-1.28) | <0.0001 |
|  |  |  |
| Hospital admission in 90 days prior to stroke admission | 1.75 (1.69-1.81) | <0.0001 |
| Prior TIA | 1.34 (1.25-1.44) | <0.0001 |
| In-hospital stroke | 1.79 (1.69-1.92) | <0.0001 |
| Intracerebral haemorrhage | 0.97 (0.92-1.02) | 0.2459 |
|  |  |  |
| *Pre-stroke Charlson Comorbidity Index (CCI)* |  |  |
| 0 | 1.00 (Ref) |  |
| 1 | 1.46 (1.40-1.53) | <0.0001 |
| 2 | 1.65 (1.58-1.73) | <0.0001 |
| >3 | 2.24 (2.15-2.34) | <0.0001 |
|  |  |  |
| Atrial fibrillation diagnosed at time of stroke admission | 1.25 (1.21-1.30) | <0.0001 |
| Hypertension | 1.39 (1.35-1.44) | <0.0001 |
| Depression | 1.32 (1.27-1.36) | <0.0001 |
|  |  |  |
| *Pre-stroke frailty index category* |  |  |
| None | 1.00 (Ref) |  |
| Mild | 1.71 (1.64-1.78) | <0.0001 |
| Moderate | 2.05 (1.96-2.15) | <0.0001 |
| Severe | 2.44 (2.24-2.66) | <0.0001 |
|  |  |  |
| *Medication prior to stroke admission* |  |  |
| Antihypertensives | 1.39 (1.35-1.44) | <0.0001 |
| Statins | 1.28 (1.24-1.33) | <0.0001 |
| Antiplatelets | 1.40 (1.35-1.44) | <0.0001 |
| Anticoagulants | 1.42 (1.34-1.49) | <0.0001 |
|  |  |  |
| Length of stay in hospital >10 days | 1.42 (1.38-1.47) | <0.0001 |
|  |  |  |
| Discharged to usual place of residence | 0.93 (0.89-0.97) | 0.0003 |
|  |  |  |
| Complete stroke care bundle | 0.96 (0.93-0.99) | 0.0160 |

Table S5. Multivariable adjusted Hazard Ratios (HR) for patient characteristics from a competing risks analysis for time to first unscheduled admission during one-year follow-up

|  | Adjusted HR  (95% CI) | P-value |
| --- | --- | --- |
| Age, years at admission |  |  |
| <50 | 1.00 (Ref) |  |
| 50-59 | 0.97 (0.89-1.06) | 0.5238 |
| 60-69 | 0.97 (0.89-1.05) | 0.4182 |
| 70-79 | 1.07 (0.99-1.15) | 0.1134 |
| >80 | 1.16 (1.07-1.26) | 0.0005 |
|  |  |  |
| Female | 1.02 (0.99-1.06) | 0.2493 |
|  |  |  |
| *Scottish Index of Multiple Deprivation (SIMD)* |  |  |
| SIMD 1 (most deprived) | 1.00 (Ref) |  |
| SIMD2 | 0.95 (0.91-0.99) | 0.0267 |
| SIMD 3 | 0.85 (0.81-0.90) | <0.0001 |
| SIMD 4 | 0.85 (0.80-0.89) | <0.0001 |
| SIMD 5 (least deprived) | 0.78 (0.74-0.82) | <0.0001 |
|  |  |  |
| *Urban-Rural classification* |  |  |
| Large Urban Areas | 1.00 (Ref) |  |
| Other Urban Areas | 1.06 (1.02-1.10) | 0.0029 |
| Accessible Small Towns | 1.05 (0.99-1.12) | 0.1215 |
| Remote Small Towns | 1.10 (1.01-1.20) | 0.0231 |
| Accessible Rural Areas | 0.99 (0.94-1.06) | 0.8672 |
| Remote Rural Areas | 1.02 (0.95-1.10) | 0.5414 |
|  |  |  |
| Independent pre-stroke | 0.99 (0.95-1.05) | 0.9094 |
| Living alone pre-stroke | 1.09 (1.05-1.12) | <0.0001 |
|  |  |  |
| *Stroke severity* |  |  |
| 0 (least severe) | 1.00 (Ref) |  |
| 1 | 1.11 (1.06-1.15) | <0.0001 |
| 2 | 1.08 (1.03-1.13) | 0.0025 |
| 3 | 1.08 (1.01-1.15) | 0.0331 |
| 4 (most severe) | 0.96 (0.90-1.03) | 0.2468 |
|  |  |  |
| Hospital admission in 90 days prior to stroke admission | 1.23 (1.17-1.28) | <0.0001 |
| Prior TIA | 1.06 (0.98-1.14) | 0.1567 |
| In-hospital stroke | 1.06 (0.98-1.14) | 0.1745 |
| Intracerebral haemorrhage | 0.98 (0.93-1.04) | 0.5369 |
|  |  |  |
| *Pre-stroke Charlson Comorbidity Index (CCI)* |  |  |
| 0 | 1.00 (Ref) |  |
| 1 | 1.17 (1.11-1.22) | <0.0001 |
| 2 | 1.26 (1.20-1.33) | <0.0001 |
| >3 | 1.49 (1.41-1.56) | <0.0001 |
|  |  |  |
| Atrial fibrillation diagnosed at time of stroke admission | 1.03 (0.99-1.07) | 0.2034 |
| Hypertension | 1.12 (1.03-1.23) | 0.0084 |
| Depression | 1.17 (1.12-1.21) | <0.0001 |
|  |  |  |
| *Pre-stroke frailty index category* |  |  |
| None | 1.00 (Ref) |  |
| Mild | 1.30 (1.25-1.36) | <0.0001 |
| Moderate | 1.38 (1.31-1.46) | <0.0001 |
| Severe | 1.48 (1.34-1.63) | <0.0001 |
|  |  |  |
| *Medication prior to stroke admission* |  |  |
| Antihypertensives | 0.98 (0.91-1.07) | 0.6807 |
| Statins | 1.01 (0.97-1.05) | 0.5745 |
| Antiplatelets | 1.13 (1.09-1.18) | <0.0001 |
| Anticoagulants | 1.14 (1.07-1.21) | <0.0001 |
|  |  |  |
| Length of stay in hospital >10 days | 1.28 (1.23-1.33) | <0.0001 |
|  |  |  |
| Discharged to usual place of residence | 1.23 (1.17-1.28) | <0.0001 |
|  |  |  |
| Complete stroke care bundle | 0.99 (0.96-1.03) | 0.8220 |

Supplementary References

1. Goldstein LB, Samsa GP, Matchar DB, et al. Charlson Index comorbidity adjustment for ischemic stroke outcome studies. *Stroke* 2004; 35: 1941–1945.
2. Gilbert T, Neuburger J, Kraindler J, et al. Development and validation of a Hospital Frailty Risk Score focusing on older people in acute care settings using electronic hospital records: an observational study. *The Lancet*, 2018; 391: 1775–1782.
3. Information Services Division Assessment of SMR01 Data 2010-2011. Scotland Report, May 2012, ISD Scotland, 2012. NHS NSS, Edinburgh, UK.
4. The Scottish Government, 2018a. Scottish Index of Multiple Deprivation. <http://www.gov.scot/Topics/Statistics/SIMD>.
5. The Scottish Government, 2018. Scottish Government Urban Rural Classification. <http://www.scotland.gov.uk/Topics/Statistics/About/Methodology/UrbanRuralClassification>
6. National Records of Scotland, 2017. Quality of National Records of Scotland (NRS) Data on Deaths. NRS, Edinburgh, UK. https://www.nrscotland.gov.uk/statistics-and-data/statistics/statistics-by-theme/vital-events/deaths/deaths-background-information/quality-of-nrs-data-on-deaths
7. Counsell C, Dennis M and McDowall M. Predicting functional outcome in acute stroke: comparison of a six simple variable model with other predictive systems and informal clinical prediction. *J Neurol Neurosurg Psychiatry* 2004: 75; 401-405.
